# The role of social justice in triage revisited: a threshold conception

**DOI:** 10.1007/s11019-024-10232-9

**Published:** 2024-11-23

**Authors:** Felicitas Holzer, Nikola Biller-Andorno, Holger Baumann

**Affiliations:** 1 Institute of Biomedical Ethics and History of Medicine, University of Zuerich, Winterthurerstrasse 30, Zuerich, 8006 Switzerland; 2 Zuerich, Switzerland

**Keywords:** Triage, Emergency triage, COVID-19, Social justice, Efficiency, Lottery, Balancing, Threshold deontology, Moral costs

## Abstract

Saving as many lives as possible while ensuring equity for vulnerable groups through access to triage resources has been the dominant position since the onset of the COVID-19 pandemic in 2020. However, the exact relationship between the principles of social justice and efficiency remains a controversial and unresolved issue. In this paper, we aim to systematically distinguish between different models of this relationship and show that conceptualizing social justice as a ‘moral side-constraint’ or adopting a ‘balancing approach’ that attempt to reconcile social justice with efficiency inevitably lead to significant moral costs that require further justification. Based on this discussion, we propose a novel “threshold model” for trading-off moral costs. According to this model, the structural impact of triage must be considered in order to determine whether one opts for triage with the primary aim of efficiency or social justice. This contextualization further explains why, in some societies and circumstances, social justice can rightly be seen as the primary concern, while in other societies and circumstances, efficiency can be defended as the primary concern.

## Introduction

Saving as many lives as possible while doing justice to vulnerable groups through the access to ICU triage has been the predominant position since the onset of the COVID-19 pandemic in 2020. This has been echoed throughout several triage guidelines that regulate access to ventilators and medical resources in the ICU context, and policy documents, which contain both justice and efficiency as two separate guiding principles (Ehni et al. 2021; Vinay et al. 2021; Phillips 2020; Emanuel et al. 2020). ‘Justice’ is frequently defined as adhering to non-discrimination or equal treatment of all social groups (and often both). Considerations of efficiency relate to the maximization of some sort of benefits, such as short-term survival, or the minimization of harms (Jöbges et al. 2020; Emanuel et al. 2020).

One of the latest examples for guiding in triage has been the Council of Europe Committee of Ministers’ recommendations on “equitable access to medicinal products and medical equipment in a situation of shortage” in 2023. These recommendations emphasize principles like “non-discrimination” and “attending to systematically disadvantaged individuals and groups in matters of health”, where “disadvantages may arise from economic and social conditions, legal status, disability, chronic illness, or age”. At the same time, the signatories maintain that priority should be given to minimizing the risk of mortality and morbidity, which highlights the critical question about how the two kinds of criteria are, both in theory and practice, to be combined (COE’s Steering Committee for Human Rights in the fields of Biomedicine and Health 2023).

Applied to the context of ICU triage and the allocation of resources to individual patients in emergency situations, the recommendations stress the importance of a systematic discussion of the precise role of social justice in triage decisions (Meier 2022). While some argue that an emergency is not the place to remedy structural injustices (Vogelstein and Krishnamurthi 2023; Savulescu et al. 2020; Marks 2020), others contend that social justice should play a direct and more important role, as certain disadvantaged groups are negatively affected by common triage criteria (Schmidt 2023; Tolchin et al. 2020). As a possible compromise, one can imagine that social justice serves as a moral side constraint, or that both principles can be ‘balanced’ against each other, as suggested notably by Reid (2020) or White and Lo (2020), and as stated in many triage guidelines and policy papers (Jöbges et al. 2020).

In this article, we will present two positions: one prioritizing efficiency in triage, and the other emphasizing social justice as the primary goal. We will then discuss current proposals to define the relationship between the two principles, i.e. to introduce social justice as a moral side constraint or to ‘balance’ the concern for social justice in the COVID-19 triage context. We then point out the structure of the problems associated with both approaches and show that neglecting the goal of efficiency in favor of social justice, and vice versa, inevitably entails violating one of the two principles, which in turn entails moral costs on both sides that require a more systematic and deeper justification. We then take a step back from the controversy and make a suggestion how to rediscuss efficiency and social justice in relation to each other, depending on the specific context of triage. More specifically, we make a structural analysis of the debate and propose a framework for evaluating efficiency and social justice in relation to one another, contingent on the specific context of triage. We argue that a threshold model is more appropriate, namely that moral costs in terms of lives lost may be acceptable if the benefits of redressing social injustice are sufficiently high. This leads us to suggest that discussions about the relation between social justice and efficiency should strongly depend on the specific context of triage, i.e. whether we are dealing with a situation characterized by only moderate or immense structural injustices, or whether we are dealing with a pure emergency or an allocation scheme with medium- or long-term implications.

## Addressing the normative gap between efficiency and social justice

As many authors argue, the goals of efficiency and social justice inevitably conflict in triage decisions (Vogelstein and Krishnamurthi 2023; Savulescu et al. 2020). Achieving a more inclusive distribution with a focus on social justice may lead to less efficient outcomes in terms of maximizing lives saved or the inefficient use of limited resources; and conversely, opting for better outcomes in terms of lives saved will inevitably lead to increased inequities through the disproportionate exclusion of vulnerable groups. The way out of this dilemma, according to some authors, is to either choose one of these guiding principles as the primary goal of triage or to harmonize both principles. In what follows, we will present both the efficiency-based and social justice-based positions, and then explore approaches that attempt to reconcile the two principles. Specifically, we will consider arguments that treat social justice as a ‘side constraint’ and those that advocate for the ‘balancing’ of the two principles. However, as we will argue, neither of these account resolves the fundamental tension between efficiency and social justice.

### Efficiency as the primary goal of triage

Several authors and policymakers sustain the position that efficiency-based considerations ought to be the primary goal of triage and concur that ‘saving lives’ represents the only acceptable interpretation of the ‘efficiency constraint’, either for pragmatic reasons or if fairness considerations are to be sufficiently taken into account (see Viany, Baumann and Biller-Andorno 2021, Savulescu et al. 2020; Emanuel et al. 2020). 1 Notably, in most guidelines, the saving of lives is asserted as the primary concern, and efficiency is therefore regarded as the primary goal of triage (Christian et al. 2014; Vincent and Creteur 2020). The justification for this position is that there might be acceptance of maximizing the number of saved lives in disaster medicine as it is *ex ante* everyone’s own interest to enter a procedure that maximizes survivors (Lübbe 2001). In the same vein, McGuire et al. (2020) acknowledge that in the wake of a pandemic, the health care system cannot remedy the structural inequalities of social systems (Marks 2020).

Even though efficiency-related triage criteria may inherently conflict with the goal of social justice, it is nevertheless widely accepted that procedural justice serves as a crucial constraint, imposing some minimal checks on efficiency-based decisions. Other than the fostering of social justice, which is usually conceived as an imperfect duty admitting of multiple possibilities of fulfillment, there are norms of procedural fairness. This is commonly conceptualized as local fairness that finds expression in the demand for non-discrimination. Non-discrimination in the narrow sense implies that characteristics such as ability, gender, or socioeconomic status should not directly influence a triage decision for an individual patient, as they would be arbitrary and thus irrelevant to the objective of saving lives. Yet, the wider question of remedying social justice remains unaddressed by local fairness.^2^

### Social justice as the primary goal of triage

Some scholars sustain that social justice should take precedence over efficiency (Stone 2020). Often, advocates of this position not only think that persistent injustices of socially salient groups ought not be worsened, conceived as a minimum constraint, but hold that they should be remedied. According to Tolchin et al. (2021), any triage decision primarily driven by a maximization rationale is likely to result in a situation where certain socially significant groups are left in a worse condition than before the triage decision was made. This disparity arises not only due to underlying comorbidities but also because preventive healthcare measures are often less effective in socioeconomically disadvantaged groups, and transmission rates tend to be higher among them. In consequence, they sustain that societies and decision-makers bear a moral duty for proactive efforts to impede that disadvantages can accumulate within socially deprived groups and exacerbate existing disparities.

Several different normative triage models are discussed as ‘fitting’ the demand for social justice as the primary goal, while efficiency remains a subordinate aim. One widely discussed normative proposal emphasizes the principle of equality, namely, the use of a lottery system for triage decisions in health emergencies (see Schmidt 2023; Schmidt et al. 2021; Stone 2020; Tolchin et al. 2020). Other proposals consider social justice and aim to enhance diversity in triage processes; for example, introducing a system with bonus points for the socioeconomically deprived, adding equity weights that counterbalance the privilege of the better-off, or eliminating scores (like the SOFA score) that create segregation between the advantaged and healthy and the disadvantaged and unhealthy (Schmidt 2023; Schmidt et al. 2021). Similarly, White and Lo (2020) suggest modifying triage guidelines by applying a correction factor to reduce the impact of structural inequalities on disadvantaged individuals.

Even more demanding normative solutions are conceivable. For instance, Tolchin et al. (2021) discuss the model of so-called ‘prioritarian triage,’ which involves allocating scarce resources to the sickest patients first or, alternatively, prioritizing resource allocation to minorities disproportionately affected by structural inequalities. The goal is to reduce or even eliminate the disproportionate disadvantages experienced by members of marginalized populations.^3^

The downside of this approach consists of the loss in the absolute number of lives saved, as we alluded to before, which has prompted counterarguments against giving social justice priority. Notably, when considering a broader conception of justice as ‘social justice’, Vogelstein and Krishnamurthi (2023) argue that injustices at a structural level do not necessarily imply that it is ethical to employ any means to rectify that unfairness in a triage situation.^4^ More specifically, the authors contend that the moral costs of neglecting the efficiency constraint, i.e., an increased number of absolute deaths, cannot be outweighed by arguments in favor of remedying injustices.

### Bringing the principles of social justice and efficiency together – is there a solution?

In a preliminary attempt to reconcile the principles of social justice and efficiency, one may be tempted to introduce social justice as a moral side-constraint, analogous to the non-discrimination demand previously outlined. The non-discrimination demand functions as a moral side constraint, meaning that the maximization rationale can only be applied if certain irrelevant group characteristics, such as race, disability, gender, etc., are excluded as criteria from triage. One may posit that social justice and the maximization rationale operate in a similar manner: any maximization constraint should, at a minimum, not deepen existing inequalities.

Another possible approach is that of so-called ‘balancing’. In line with the essence of many triage guidelines implemented during the COVID-19 pandemic, Reid (2020) contends that it is still possible to give justice a prominent role in triage decisions and make this aim compatible with the maximization rationale. She proposes a ‘balancing’ approach, suggesting that we can ‘balance’ values and principles of equal weight to resolve moral dilemmas without rendering the ethical dilemma ‘illusory’ in view of our commitment to compromised values (Reid 2020: 527). In this instance, it is conceivable that social justice may be regarded as an equally important goal alongside efficiency, and that it may be balanced at an equal level.

Reid (2020) more specifically argues that it seems unjustified to follow the ordering of patients under the efficiency rule if differences in survival chances are small. Most notably, she argues, “(…) a balancer might readily agree to prioritize saving the most lives if the choice is between a person who has a 10% chance of survival and a person who has a 90% chance of survival. Where the differences are smaller, it is plausible to reason that the differences in survival probability between two persons do not justify a categorical difference in treatment” (Reid 2020: 527). The fact that there are thresholds between which patients are given equal weight, regardless of the fact that they do not have the same survival probability, gives people with lower (yet sufficiently high) survival probabilities a chance to be considered in a triage decision. This means that vulnerable groups that often show lower survival probabilities are given a higher chance of being considered. As the threshold for considering patients within the treated group decreases, there is a greater likelihood of including individuals with comorbidities and other unfavorable characteristics that can adversely impact survival.

Giving social justice even more weight, White and Lo (2021) suggest operationalizing social justice by introducing parameters for social justice-based mitigating health disparities between advantaged and disadvantaged groups alongside health outcome-based criteria that aim at efficiency. More specifically, the authors suggest introducing a correction factor to reduce the impact of structural inequalities alongside the prognosis of hospital survival (White and Lo 2021, p. 292). A number of other suggestions have been put forward which are based on the same rationale.^5^

A closer examination of both the possibility of introducing social justice as a ‘side constraint’ and ‘balancing’ social justice into triage decisions reveals the following problem: The incorporation of a social justice constraint, even in its most minimal form, is not aligned with the efficiency rationale if vulnerable groups continue to bear disproportionate burdens. This persistent issue arises due to the assumption that criteria based on the efficiency rationale (such as prognosis or survival probability) correlate with the prevalence of unfavorable determinants of health within socially disadvantaged groups (Schmidt et al. 2021). If this is indeed the case, any triage process based on efficiency considerations will inevitably result in worse outcomes for certain vulnerable groups, even when applying the balancing account where small differences in survival probabilities are not being considered. In other words, if we formulate the constraint of ‘not worsening social justice’ as a deontological side constraint similar to local fairness constraints, this constraint will be violated. Reid’s version of balancing suggests that the broader social justice constraint is not being violated at all. Nevertheless, balancing may entail that the moral costs associated with social injustices are less pronounced compared to a scenario in which the efficiency principle remains unchecked. The violation of the social justice constraint is less apparent in less fine-grained decision models that opt for less efficiency, as exemplified by the model presented by Reid (2020). This is because such models offer disadvantaged groups a higher chance of being considered in a triage situation. Nevertheless, it should be noted that the objective of making the maximization rationale more ‘socially inclusive’ (by reducing its negative impact on social justice) does not imply that disadvantaged groups would necessarily have equal chances in absolute terms.

This might be different in an approach where social justice concerns are given equal or more weight alongside efficiency-based criteria. However, here the problem may be the other way round: depending on the correction factor, especially if it significantly changes the points allocated to each patient, it may significantly change the health outcome in terms of efficiency. Efficiency-based factors, such as the short-term prognosis for survival in hospitals, could be outweighed by the adjustment factors. The allocation of equity points means that disadvantaged patients with a lower chance of survival will be treated instead of a patient with a higher chance of survival, because the total priority points from both efficiency and non-efficiency criteria are lower. However, this generally runs counter to the principle of allocating resources under the efficiency constraint and implies that fewer people can be helped overall, as we will explain in more detail in the following sections (see Vogelstein and Krishnamurthi 2023).

As an important addition to this debate, it can be argued that the principle of efficiency has an inherent asymmetry compared to other principles, in particular because of its intrinsic link with the concept of triage: triage is always a process aimed at optimizing the use of resources in situations of extreme scarcity. However, a narrow interpretation of efficiency that focuses only on maximization overlooks its broader implications. So, authors who argue for a more prominent role for social justice would say that they are still committed to choosing the most efficient scenario among all options that are consistent with social justice constraints. At the same time, it is also true that giving less priority to efficiency may seem to contradict the principle of efficiency as a primary objective. After all, both efficiency and social justice can be violated to varying degrees—when we move toward one goal, the other is compromised in its absolute form. However, this does not imply that the two goals are ‘balanced’ in the sense of being ‘harmonized’ or ‘reconciled.’ Rather, it would be more accurate to say that they are traded off against each other.

## Towards a threshold model for triage decisions and the demand for social justice

We will now examine the moral implications of the outlined positions in greater depth. At this point, it is important to note that advocates of either position often criticize each other for neglecting the moral costs associated with the other position. In this context, the aim of the following sections is to develop a comprehensive account that can make sense of both notions, namely social justice and efficiency as central goals of triage. We suggest that this can be achieved through what we term a *threshold model*. We will proceed in a step-by-step manner, examining moral costs, threshold deontology, and then applying that to triage.

### Moral costs

Let us assume that we are morally obligated to mitigate health disparities resulting from social injustice between advantaged and disadvantaged ethnic or socioeconomic groups if we can do so without incurring an equal or greater moral cost. We may also assume that it is feasible to do so by introducing social justice-based triage criteria. Yet, there is another assumption needed to adhere to social justice as the primary aim of triage, which is contested by many scholars: the moral costs of social justice-based criteria, including more people to die, are outweighed by the moral positives of disparity mitigation. Let us refer to this premise as the ‘primacy of social justice’ claim.

Authors who advocate for prioritizing efficiency over social justice, like Vogelstein and Krishnamurthi (2023), argue that this foundational premise is flawed. While they concede that we can have *prima facie* an imperfect duty to remedy social injustices, they reject ‘the primacy of social justice’ claim on the grounds that the moral costs associated with prioritizing social justice, particularly in terms of equity criteria for triage decisions, do not outweigh the corresponding benefits. Furthermore, the authors assert that the obligation to rectify injustice hinges on the direct causal link to the injustice itself. If those responsible for remedying injustice are not the primary cause, there is no moral justification for sacrificing the well-being of innocent individuals to address the injustice.^6^ This ethical dilemma is accentuated in the context of triage during a pandemic, where individuals outside vulnerable groups might receive fewer life-saving resources, despite allocating resources to them potentially saves a higher number of patients.

Nevertheless, an objection may arise suggesting that the moral costs of remedying injustices are less significant than initially presumed. For instance, we might assume that numbers matter.^7^ We could imagine a scenario, where opting for a social justice policy would result in only a negligible net loss of lives – for example, causing 1001 deaths instead of 1000 (Vogelstein and Krishnamurthi 2023, p. 235). However, critics like Vogelstein and Krishnamurthi (2023) argue that this reasoning lacks moral force, contending that there is still no compelling reason why rectifying injustices should be preferable when there is a net loss in lives saved.^8^

However, a further analysis could be conducted by examining the moral costs that are directly caused by the failure to address social injustices. As previously discussed, efficiency-based policies can exacerbate existing injustices, leading to moral concerns beyond intrinsic moral worries. Social injustice can impose substantial costs on disadvantaged groups, affecting their well-being, longevity, and eroding public trust and social cohesion (Elias and Paradies 2021; Glyn and Miliband 1994). It is of significant importance to note that these discrepancies between social groups can accumulate over time, resulting in higher moral costs. Without undertaking a detailed cost analysis, it seems implausible to categorically reject the claim that social justice should be prioritized. Otherwise, we would be forced to assume that the net loss in saved lives always produces more significant moral costs compared to the costs of increased social injustice, which seems implausible regardless of the precise context. It is uncertain whether there is a point at which the number of deaths becomes acceptable in order to avoid the greater evil of substantial and continuous injustices.

### Threshold deontology for considering moral costs

The term ‘threshold deontology’ has regained significance in the field of moral philosophy and is frequently associated with acts that are deemed morally wrong despite yielding a net positive balance of consequences (Alexander 2000). Wonnell (2011) defines this position as the belief that there are certain actions that are forbidden even if their consequences are desirable, unless those consequences are *extremely* desirable. Moreover, the term is often associated with acts that are morally wrong despite producing a net positive balance of consequences. However, if the positive balance of consequences becomes sufficiently great, then one is morally permitted, and perhaps required, to engage in those acts that are otherwise morally prohibited. The underlying premise is that consequences are always taken into account, even when they are below a certain threshold. However, the moral costs associated with consequences may be weighed differently, such that the rightness of an action can be overridden once a certain threshold is crossed. Moreover, threshold deontologists argue that moral constraints are never absolute and that the moral cost of an action is not necessarily proportional to the action itself.

This account may not be helpful in resolving the practical problem of determining when such a threshold commences, but it can nevertheless offer an account to describe a theoretical problem of determining why we think that a certain action, which is *pro tanto* right, can become wrong if its moral costs become too high.

### A threshold model for triage decisions

Defenders of the efficiency-based view may argue that in moderately unjust circumstances the positive effects of social justice criteria cannot outweigh by large the moral costs of more people dying. In the same vein, we may then argue that social justice understood as a deontological constraint, *pro tanto*, cannot be violated up to a certain threshold, but beyond that threshold, this may be morally permissible and even required. In other terms, the moral pressure on agents in a triage situation to save as many lives as possible may indeed override the duty to mitigate or not to exacerbate social inequalities. Threshold deontology implies that there is a certain ‘break point’ or ‘threshold’ at which deontological constraints can be violated, making it morally permissible. Unlike balancing, threshold deontology holds that one of two normative principles becomes more significant at this threshold, which depends on a specific context.

Let’s return to the ‘primacy of social justice’ claim and the moral costs of implementing triage criteria that account for social justice. One might argue that the structure of the ‘primacy of social justice’ claim resembles a threshold conception. While the ‘primacy of social justice’ claim might be flawed in a moderately unjust society, as put forward by many defenders of the efficiency-based view, it could be deemed morally righteous in a deeply unjust society characterized by high disparity and high moral costs of ignoring the social justice concern. Of course, this does not presume that thresholds need to be breaking points implying an absolute change in triage goals. We rather suggest that efficiency may gain importance with the degree of severeness and acuteness of an emergency; and social justice may gain importance with the severeness of structural injustices.

### Contextualizing triage

Applying the insights from the past sections, we hold that the choice of a normative model for triage, either guided by efficiency considerations or social justice, depends on the precise context. Differently put, whether it is considered adequate that moral costs of social justice-based criteria are outweighed by the positives of disparity mitigation depends on the circumstances of justice (using the Rawlsian term). To be more precise, it is not only about the fact that the positives of disparity mitigation outweigh the moral costs of more people dying, but also about the fact that justice mitigation might be extremely desirable in certain circumstances. So, let us take a closer look at the following two scenarios.

First, as the comparison of triage guidelines suggests, there are countries in which health disparities are great and strongly correlate with ethnicity and income status. Triage then, one might argue, cannot merely be seen as a process of local fairness but as a phenomenon that itself is linked to some sort of background structure and where social justice concerns become most visible. In other words, we could think of triage as embedded in a context of structural injustice, as only one manifestation of a long series of local allocation schemes that, in a sum, are a result of and at the same time contribute to structural injustice, including outcome equality and socioeconomic standing. This view implying an inherent connection between local and global justice considerations has previously been discussed, most notably by Young (2008) who argues that that local structures and injustice cannot be understood without turning our eyes towards structural injustice (Young 2008). Scholars like Tolchin et al. (2021) argue that this recognition calls for local rules to contribute to the development of more just social structures.

On the other hand, in a moderately unjust society where structural disadvantages might still occur but not greatly impact healthcare access, and, speaking with Daniels, the ‘normal functioning’ of people, triage may mainly resemble a procedural allocation scheme, where the costs of social injustice might be negligible. That is, there is most likely no long-term impact attached to the moral costs in terms of worsened injustice, and minority groups are not systematically affected by the negative consequences of an efficiency-driven triage. To illustrate both positions, the U.S. discourse mainly highlighted severe structural injustices leading to the systematic disadvantage of minority groups in the triage processes implemented in the wake of the COVID-19 pandemic, while the discourse in more moderately unjust countries with fewer income, ethnicity, and health-related disparities mainly focused on the efficiency concern.^9^

Second, whether the ‘primacy of social justice’ claim ought to be rejected or not might also depend on how we conceive a pandemic. For the sake of the argument, let’s consider different scenarios.

There is the possibility that triage is conceived as a pure state of emergency, such as in the wake of the COVID-19 pandemic, similar to the situation in which people are triaged in car accidents, that can randomly affect people regardless of their socio-economic status. In such a context of emergency, many authors argue that health care systems ought to primarily keep a system running instead of remedying structural inequalities (Marks 2020; McGuire et al. 2020). Here, a general transfer of this idea to “everyday medicine” is not plausible if we assume to distribute resources such that everyone has a fair chance of accessing medical care, given that we have significantly more *ex ante* knowledge about the outcome of the maximizing rule in “normal circumstances”, especially concerning the vulnerable. Yet, even in the situation of an emergency, the comparison to car accidents may not fully capture all complexities. Some healthcare systems are sometimes barely prepared for the specific circumstances of a pandemic, and health disparities may worsen due to a lack of capacity to adequately treat certain patient groups with special needs.

Therefore, we might perceive pandemics differently from accidents, as they can have broader implications. They could be seen as foreseeable occurrences in which it can be anticipated that vulnerable and socially significant groups may bear disadvantages. Differently put, triage may not only harm patients at an individual level but can also perpetuate systemic injustices. This reasoning gains further strength when triage leaves the state of emergency and transitions into a mid- and/or long-term perspective with implications of substantive social costs for vulnerable groups and social cohesion, especially when there is no immediate need to act drastically facing an extreme shortage of medical resources but a relative shortage, as seen in the aftermath of the acute pandemic phase.^10^

In this third scenario, a pandemic can be considered as a continued state. Tolchin et al. (2021), for instance, argue that it might be imperative during a pandemic to adopt a long-term view at a certain point that considers future epidemic waves, as an accumulation of local injustices over time can worsen social justice. One aim of ethical triage in this context ought to be the avoidance or even remedying of social costs produced in an emergency by promoting “social cohesion and healing after a crisis (Solomon et al. 2020; Marks 2020).

In view of the outlined scenarios, structural impact might become the primary concern in two ways: when triage occurs in extremely unjust societies and when it leaves the state of emergency and becomes a local process with a considerable structural impact. To support this view and address potential critique, we consider it essential to examine the empirical evidence on how triage decisions during the COVID-19 pandemic have contributed to structural disparities between groups or whether the lack of consideration for vulnerable groups (for the sake of efficiency) has remained a “local injustice” without significant broader implications for the background structure.

## Conclusion

The purpose of this paper was twofold. The first aim was to present the various positions on the triage principles of efficiency and social justice, and to show that attempts to “balance” these ethical principles inevitably result in a violation of one of the two principles, with significant moral costs. The question then arises as to which moral costs resulting from this violation of principles should be incurred in the context of a trade-off. As we discussed in Sect. 3, it appears that a reasonable course of action would be to trade off the moral costs associated with violating the principle of social justice under a “threshold model”. This would entail accepting a greater number of deaths as a moral option only if the reduction of injustices becomes a highly desirable goal.

Thus, we propose that any debate about social justice in the triage context should consider the context in which efficiency-related costs and costs stemming from the exacerbation of social injustices occur. In moderately unjust societies or emergencies with a high mortality rate, efficiency becomes a primary concern. However, in deeply unjust contexts or ongoing situations where public health isn’t immediately threatened, social justice might be paramount. This is especially evident in the discourse in the US, where socioeconomic and historical inequality is a major problem and gains importance as we approach an endemic phase of a health crisis (Schmidt et al. 2021; Schmidt 2023; White and Lo 2020).

That said, our bottom line is that triage needs to be contextualized. In practical terms, this could mean that triage guidelines and public policies should be explicit about the described trade-off and the consequences of it, in order to facilitate transparent triage decisions. Furthermore, it is recommended that authors of guidelines and policy makers provide clarity about this contextualization and explicitly state the consequences of these considerations for triage decisions. This may include the choice of triage criteria and their operationalization in guidelines.

Yet, the objective of this paper has been to elucidate the framework for discussing the role of social justice. We have not delved into the specific implications this may have for triage criteria. While one might argue that in deeply unjust societies, triage through mechanisms such as, for instance, a lottery could be a viable approach, this is a topic that warrants further exploration. In this paper, we do not take a stance on the practical ramifications but emphasize that the discourse on social justice must be undertaken. This could be achieved through a dynamic approach that evolves over time and can be adapted to the concrete circumstances. One aspect of this clarification may be to determine whether there are residual duties remaining after a pandemic that has primarily focused on the saving of lives. This could include duties to remedy the injustices and incurred moral costs of triage.

## Footnotes

1 In contrast to the policy discourse, the ethical debate has been led in a more controversial way; based on utilitarist and pragmatist perspectives, as well as deontological considerations on the equal value of life. In some sense “saving lives” represents a pragmatic approach to triage, even though authors admit there could be also other crtieria put forward. For reasons of the stipulations with the scope of this paper, we will not present the nuances of these debates.

2 This analysis draws on a narrow understanding of justice as local fairness norm, as e.g. presented in and conceptualized by Eidelson (2015) and Lippert-Rasmussen (2006). Eidelson, in particular, understands the concept of “direct discrimination” in a narrow sense as the absence of arbitrary unequal treatment and the absence of stereotypical attribution of characteristics based on group membership.

3 Indeed, article 6.1 of the “recommendations on the equitable access to medicinal products and medical equipment in a situation of shortage” and particularly the formulation that “the severity of the health condition of the individual concerned and the healthcare needs” should be taken into account for prioritization could be interpreted in the sense of a prioritatian model (COE’s Steering Committee for Human Rights in the fields of Biomedicine and Health 2023).

4 The authors’ reflections address, among other things the remedying of disparities in COVID outcomes that are caused by underlying unjust social conditions. Here, we delineate and critique those arguments. We argue that not only are such arguments unconvincing but also that there are compelling moral reasons to reject the sort of equity-included allocation schemes at issue. In several case scenarios making allusion to the trolley thought experiment, they show that remedying structural injustices cannot be demanded in triage decisions, as its positive effects do not outweigh the moral costs caused, including that more people die (see Volgelstein and Krishnamurthi 2023, p. 332ff).

5 Many approaches are particularly designed for the United States, where inequities are notably severe (see Schmidt et al. 2021; White and Lo, 2021).

6 For the sake of the stipulations within the scope of this article, we will not pursue this line of discussion further. However, there is an extensive debate on rectifying duties in the literature.

7 We acknowledge that there is an emerging literature attempting to quantify the magnitude of trade-offs in terms of numbers and health outcomes in ICU triage (see, for example, Bhavani et al. 2021). Here, additionally to theoretical elaborations, empirical studies attempt to assess the impact of different triage strategies.”

8 The authors base their argument on the assumption that there is no compelling reason to consider rectifying duties as long as no direct causal link can be established between an agent and the occurrence of injustice. Rectifying duties would only apply to those who stand in a direct causal relationship to the circumstances that ought then to be rectified. However, it is true that this position is highly contested by structural perspectives on injustice, as defended by Young (2008). Yet, as we previously alluded, we will not rely on the discussion around rectifying duties for further argumentation but will instead adopt a prospective view to argue why social injustices need to be rectified.

9 Also see [blinded] on the context of triage in Switzerland, where efficiency-based consideration and arguments dominated in the SAMS (2021) triage guidelines (see https://www.sams.ch/en/Ethics/Topics-A-to-Z/Intensive-care-medicine/Triage-intensive-care-medicine.html*).*

10 Yet, we acknowledge the possible critique that triage is about emergencies and does not necessarily imply a mid- and long-term perspective. Yet, we would like to set against this that triage guidelines written for the emergency context are still effective for the endemic context. Also, the transition between emergency and endemic state are subtle, and often, there is not clear break-off point.
