# Correction: The role of social justice in triage revisited: a threshold conception

**DOI:** 10.1007/s11019-025-10250-1

**Published:** 2025-01-25

**Authors:** Felicitas Holzer, Nikola Biller-Andorno, Holger Baumann

**Affiliations:** 1 Institute of Biomedical Ethics and History of Medicine, University of Zuerich, Winterthurerstrasse 30, Zuerich, 8006 Switzerland; 2 Zuerich, Switzerland

Medicine, Health Care and Philosophy

10.1007/s11019-024-10232-9

The Funding information was incorrect in this article and should have read ‘Open access funding provided by University of Zurich. Funded by the Swiss National Science Foun­dation, grant number 207672’ instead of ‘Open access funding provided by University of Zurich’.

The original article has been corrected.

